# Adipose-derived exosomal miR-210/92a cluster inhibits adipose browning via the FGFR-1 signaling pathway in high-altitude hypoxia

**DOI:** 10.1038/s41598-020-71345-8

**Published:** 2020-09-01

**Authors:** Yifan Zhang, Kang Song, Gang Qi, Ranran Yan, Yanqing Yang, Yan Li, Shunjuan Wang, Zhenzhong Bai, Ri-li Ge

**Affiliations:** 1 Research Center for High Altitude Medicine, Qinghai University Medical College, Qinghai, 810001 Xining People’s Republic of China; 2 Key Laboratory of High-Altitude Medicine (Qinghai University), Ministry of Education, Qinghai, 810001 Xining People’s Republic of China; 3 Key Laboratory for Application of High-Altitude Medicine in Qinghai Province, Qinghai, 810001 Xining People’s Republic of China; 4 Qinghai Provincial People’s Hospital, Qinghai, 810007 Xining People’s Republic of China

**Keywords:** Fat metabolism, Endocrinology

## Abstract

Cold and hypoxia are critical drivers of adaptation to high altitudes. Organisms at high altitudes have adapted to maximize the efficiency of oxygen utilization and are less prone to obesity and diabetes than those at low altitudes. Brown adipose tissue (BAT) dissipates energy in the form of heat in both humans and rodents; it also serves to regulate metabolism to curb obesity. However, the role of BAT in high-altitude populations is poorly understood. Serum exosomes can be easily obtained, enabling the study of BAT functions and identification of biomarkers in serum exosomes, both of which contribute to understanding the role of BAT in high-altitude populations. ^18^F-Fluorodeoxyglucose (^18^F-FDG) positron emission tomography integrated with computed tomography (PET/CT) is the gold standard for studying BAT in human adults. Here, we studied BAT in healthy high-altitude populations via PET/CT and serum exosomal microRNAs (miRNAs). The observations were validated in mouse tissues and demonstrated that high-altitude hypoxia activated BAT through attenuated white adipose tissue (WAT) secreted exosomal miR-210/92a, which enhanced the FGFR-1 expression in BAT.

## Introduction

Hypoxia exerts profound systemic effects on metabolism. It is implicated in a broad spectrum of metabolic disorders, such as obesity and diabetes^1^. However, the effects of hypobaric hypoxia on adipose tissues is poorly understood. But the mechanisms of high-altitude adaptation have been extensively investigated^2–4^. These mechanisms include enhanced oxygen delivery, efficient consumption of limited oxygen through increased RBC production^5,6^ and reprogramming of both global^7^ and tissue-specific metabolic processes, such as decreases in muscle oxidative phosphorylation and fatty acid oxidation but increases in cellular glycolysis and mitochondrial coupling efficiency^8,9^. These adaptations have enabled populations dwelling long-term at high altitudes to survive. More importantly, these populations have a lower risk of diabetes, obesity, and other metabolic syndromes than sea level populations^10–12^.

Obesity can be caused by excessive accumulation of adipose tissue, which in turn induces hyperlipidemia, cardiovascular diseases, and metabolic syndromes^13,14^. Increased BAT activity is associated with the enhanced heat generation and energy consumption, which in turn confers beneficial effects on adipose development, glucose metabolism and against obesity. Both pharmacological and physiological stimulation can be applied in preclinical approaches to promote BAT activity in human and rodent models. Therefore, revealing the mechanisms modulating BAT development will provide new insights for the prevention and treatment of these metabolic diseases. However, given their limited effects and unclarified side effects, reliable methods to achieve this goal will not be quickly identified. Interestingly, as we have previously reported, native mammals living on the Qinghai-Tibetan Plateau exhibit increased BAT activity. Therefore, we hypothesize that increased BAT activity could be a new adaptation strategy for high-altitude hypoxia^15,16^.

Exosomes are small circular lipid vesicles with a diameter of 40–100 nm and unique markers on their membranes. They can be abundantly secreted from adipose tissues and contain many microRNAs (miRNAs) that are released into plasma and taken up by the neighboring or remote recipient cells to modulate multiple biofunctions. Therefore, exosomes have been associated with new cell–cell communication patterns^17^. The involvement of circular miRNAs from exosomes in multiple metabolic modulations, such as insulin resistance and glucose metabolism, along with BAT activation in humans and rodents, has been widely described^18^. More interestingly, exosomal miRNAs play important roles as both endocrines signaling molecules and disease markers. For example, miR-92a is associated with high-density lipoprotein (HDL) components that indicate increased risk of CVD^19^. However, little is known about BAT activity in high-altitude populations. In this study, we used positron emission tomography integrated with computed tomography (PET/CT) scanning in high-altitude populations to clarify the global BAT amount. In addition, the exosomes were isolated to identify variations in the expression of candidate exosomal miRNAs. Notably, high-altitude-raised mice also showed enhanced BAT activity and similar miRNA alterations in exosomes. Moreover, the exosomal miRNAs originated from WAT and activated BAT through enhanced the FGFR-1, which demonstrated that WAT plays the leading role in the high BAT activity under chronic hypoxia exposure.

## Results

### High-altitude hypoxia increased BAT activity in humans and mice

**Figure 1:**
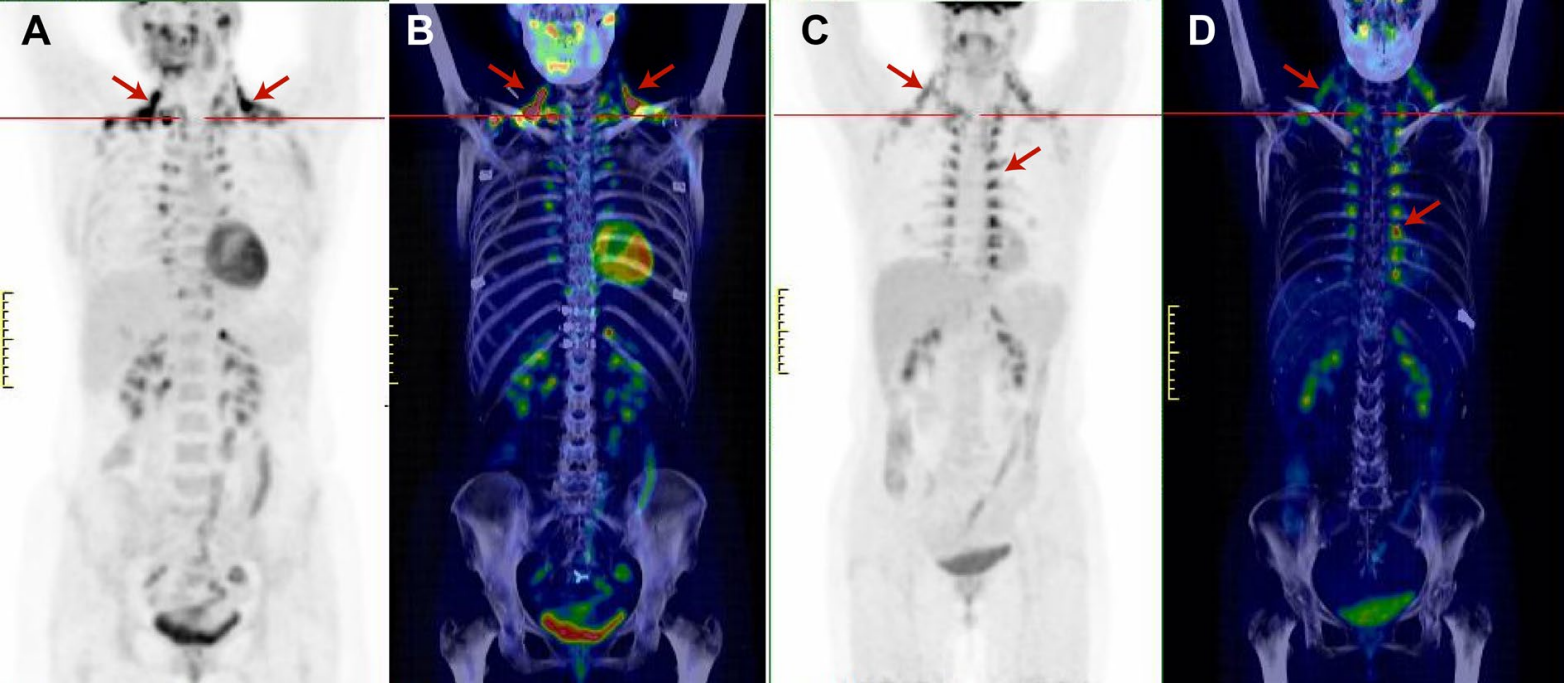
Anterior maximum intensity projection visualization and 3D mimic images of BAT ^18^F-FDG uptake in the high-BAT group (**A**, **B**) and low-BAT group (**C**, **D**). The arrows indicate the paravertebral-intercostal and mediastinal regions in the neck and supraclavicular/axillary regions.

We found 21 scans (21 healthy volunteers) with activated BAT among 239 evaluated ^18^F-fluorodeoxyglucose (^18^F-FDG) PET/CT scans. In previous reports^20^, five anatomical sites have been used to assess BAT—subphrenic, paravertebral, mediastinal, cervical, and supraclavicular. We selected volunteers evenly distributed by sex, age, BMI, and season. After 6 h of fasting, all volunteers exhibited various levels of ^18^F-FDG uptake in the above five locations after injection of ^18^F-FDG and exposure to 4 °C. We defined the group of individuals with a maximum standard uptake value (SUVmax) greater than 4.5 as the high-BAT group, and the group of individuals with an SUVmax less than 4.5 as the low-BAT group, as shown in Fig. 1. The general characteristics of the subjects are shown in Table 1. The values of BMI, body weight and the other general parameters did not significantly differ between the two cohorts. Interestingly, we found a significant difference in the residence altitude between the two cohorts. Next, we defined the group of individuals residing at an altitude above 3,000 m as the high-altitude group and the group of individuals residing at an altitude below 3,000 m as the low-altitude group. The ^18^F-FDG SUVmax of BAT in the two cohorts was significantly different (*p* = 0.021) 6.831 ± 1.08 and 3.872 ± 0.32, respectively.

**Table 1:** General characteristics of high-altitude adaptation subjects.

	Total (n = 21)	Low BAT (n = 10)	High BAT (n = 11)	p
Male/females	11/10	4/6	7/4	–
Age (years)	34.67 ± 2.415	35.10 ± 2.23	34.27 ± 2.61	0.45
Height (m)	1.701 ± 0.076	1.731 ± 0.808	1.68 ± 0.653	0.23
Altitude	3,111 ± 862	2,666 ± 228	3,516 ± 240	0.02*
Weight (kg)	66.57 ± 8.721	67.90 ± 9.712	65.36 ± 7.99	0.85
BMI (kg/m^2^)	22.89 ± 1.599	22.54 ± 1.73	23.19 ± 1.47	0.32
Waist/hip	0.834 ± 0.099	0.833 ± 0.115	0.834 ± 0.89	0.96
Fasting glucose (mmol/l)	5.162 ± 0.511	5.07 ± 0.54	5.245 ± 0.49	0.45
RBC (10^12^/l)	4.729 ± 0.426	4.780 ± 0.40	4.682 ± 0.46	0.61
HGB (g/l)	143.9 ± 15.85	145.9 ± 14.8	142.1 ± 17.3	0.59
TCh (mmol/l)	4.148 ± 0.539	3.95 ± 0.59	4.33 ± 0.44	0.11
TG (mmol/l)	1.754 ± 0.984	1.53 ± 1.04	1.96 ± 0.93	0.34
HDL-C (mmol/l)	0.998 ± 0.151	0.98 ± 0.12	1.01 ± 0.18	0.61
LDL-C (mmol/l)	2.916 ± 0.631	2.85 ± 0.71	2.97 ± 0.58	0.67
Apo-A1 (g/l)	1.300 ± 0.083	1.27 ± 0.05	1.33 ± 0.10	0.12
Apo-B (g/l)	1.370 ± 0.765	1.51 ± 0.73	1.25 ± 0.81	0.46
HBA1C (%)	5.168 ± 0.366	5.14 ± 0.38	5.19 ± 0.37	0.74
C-peptide (ng/ml)	2.532 ± 0.688	2.43 ± 0.71	2.63 ± 0.69	0.52
FINS (μU/ml)	9.775 ± 2.196	9.47 ± 2.20	10.05 ± 2.26	0.55
miR-210 (FC*)	0.854 ± 0.393	1.09 ± 0.41	0.63 ± 0.21	0.06*
miR-92a (FC)	0.747 ± 0.404	1.08 ± 0.31	0.45 ± 0.18	0.00*
SUV mean	3.161 ± 1.799	2.04 ± 0.56	4.184 ± 1.94	0.04*
SUV max	5.422 ± 3.031	3.36 ± 0.67	7.291 ± 3.14	0.02*

*BMI* body mass index, *Tch* total cholesterol, *TG* triglyceride, *HBA1C* glycated hemoglobin, *HDL-C* high density lipoprotein cholesterol, *LDL-C* low density lipoprotein cholesterol, *Apo-A1* Apolipoprotein A1, *Apo-B* apolipoprotein B, *FINS* fasting insulin, *SUV* standard uptake value.

*FC fold change.

**Figure 2:**
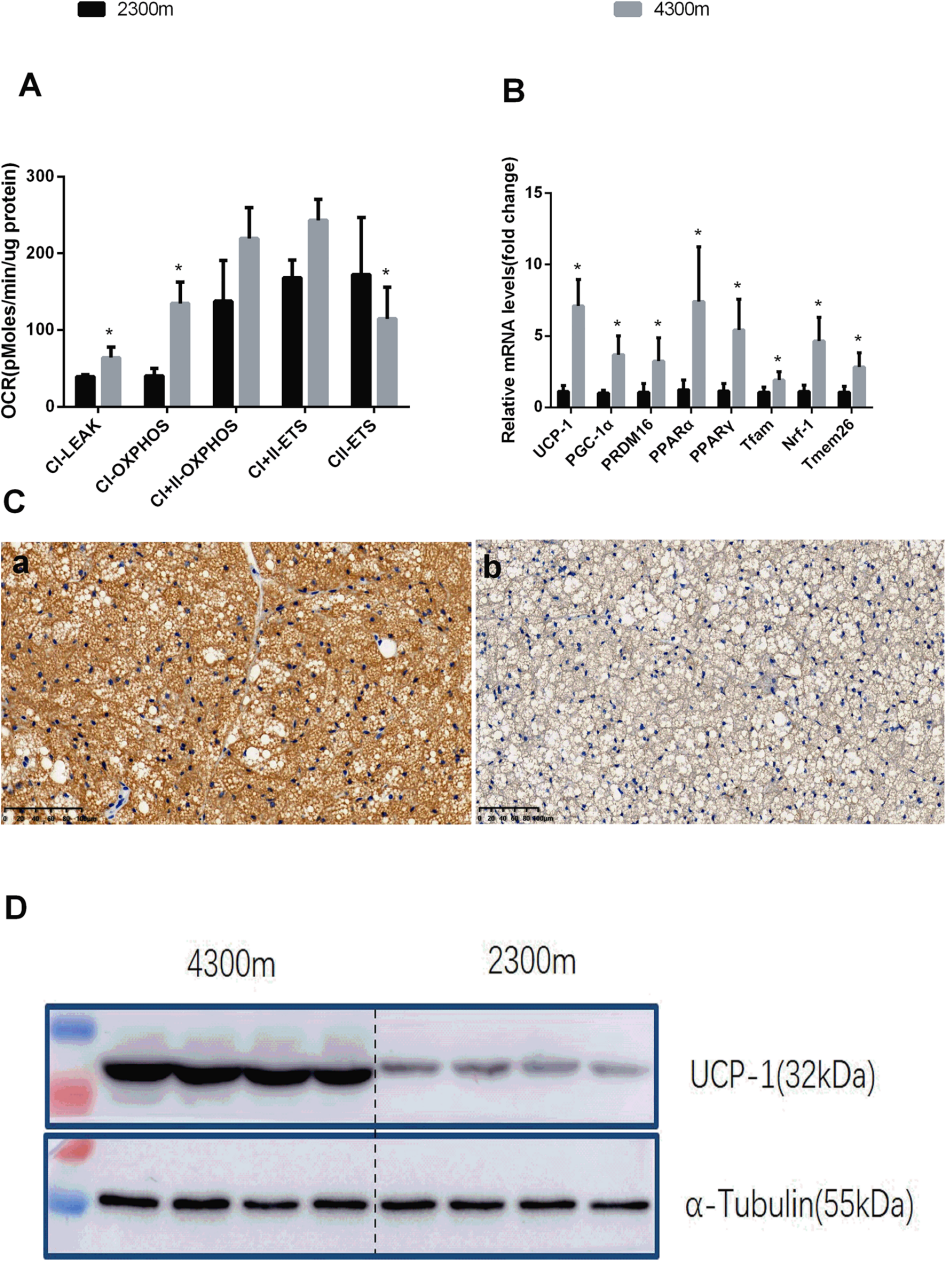
(**A**) BAT mitochondrial respiratory capacity in 2,300 m- and 4,300 m-raised mice (n = 5 per group; p ≤ 0.05 was statistically significant). The 2,300 m-raised group was used the control. CI-LEAK is the state 4 respiration rate of respiratory chain complex I. CI-OXPHOS is the mitochondrial respiratory chain complex I state 3 respiration rate, CI + II-OXPHOS is the respiratory chain complexes I + II state 3 respiration rate, CI + II-ETS is the uncoupling ability, and CII-ETS is the electron transfer capability of respiratory chain complex II. (**B**) Brown adipose-selective genes’ relative expression levels in BAT from mice raised at 2,300 m or 4,300 m for 8 weeks. The results were obtained by qPCR analysis. The data were normalized to the levels of 18S rRNA in 2,300 m group mice (n = 6 per group; *p ≤ 0.05, Student’s *t* test.) (**C**) Representative immunohistochemical results in BAT from 2,300 m- and 4,300 m-raised mice [(**A**) 4,300 m group BAT. (**B**) 2,300 m group BAT]. Magnification, × 200. (**D**) Protein expression levels of UCP-1 in BAT from 2,300 m- and 4,300 m-raised mice.

To validate the population study results, C57BL-6J mice were raised at gradient altitudes of 2,300 m and 4,300 m under the same conditions. We found increases in BAT mitochondrial activity in terms of the oxygen consumption rates (OCRs) in complexes I and II; in addition, the PCR results showed increased levels of browning-selective genes such as *Ucp-1*, *Pgc-1α* and *Prdm16*. Furthermore, the Western blot analysis and immunohistochemical staining results showed increased expression of UCP-1 in 4,300 m-raised mice (Fig. 2).

### Expression of the miR-210/92a cluster in serum exosomes was negatively correlated with BAT activity

**Figure 3:**
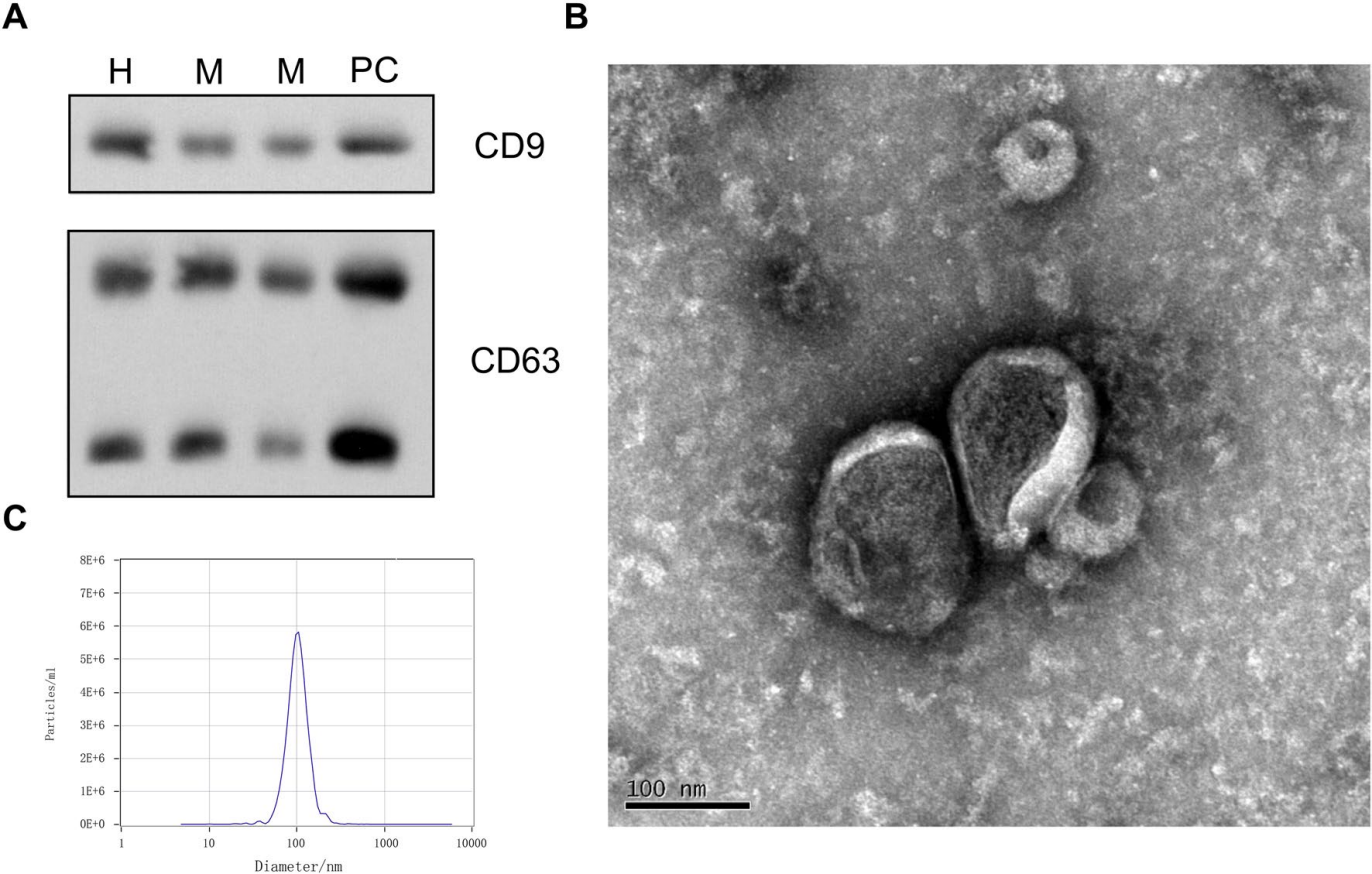
(**A**) Qualitative Western blot showing the exosomal expression of the exosome marker proteins CD63 and CD9 (PC is for positive control, M is mouse sample, H is human sample). (**B**) Electron micrographs of exosomes in serum (left scale bar, 100 nm). (**C**) The particle size distribution (PSD) of exosomes was measured by nanoparticle tracking analysis; the concentration and zeta potential, by analysis of the particle traces.

The purity of the exosomes was assessed before miRNA analysis. Initially, the exosomal protein markers CD9 and CD63 were detected by Western blotting. Then, the exosomes were observed with transmission electron microscopy (TEM), and the exosomal particle sizes and concentration were measured through nanoparticle tracking analysis (NTA). The results are shown in Fig. 3.

**Figure 4:**
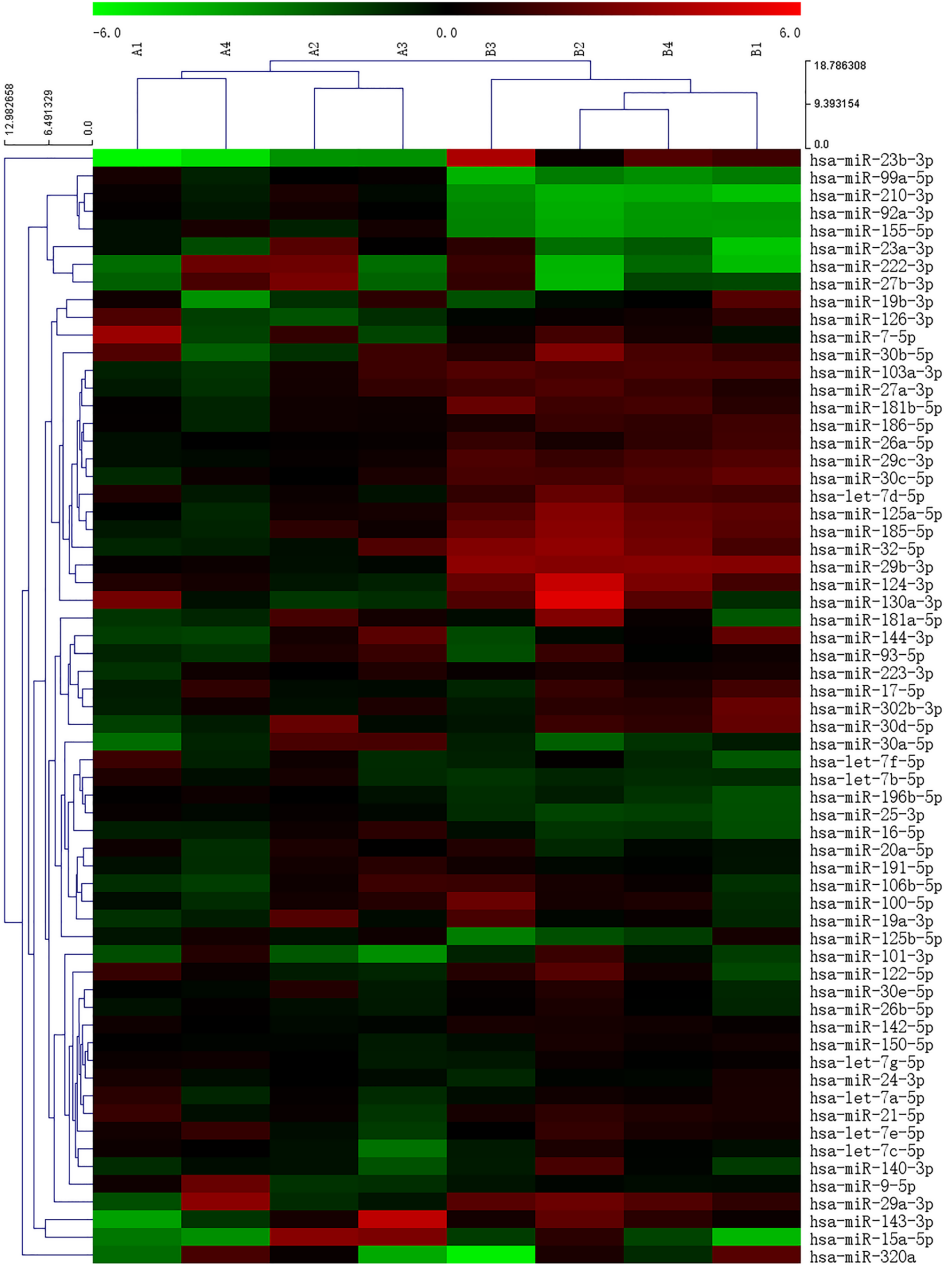
Heatmap of exosomal miRNAs differentially expressed between the high-BAT and low-BAT groups. Hierarchical clustering analysis of the 84 commonly expressed miRNAs by the Euclidean distance method. Each row represents one miRNA, and each column represents one tissue sample. The relative miRNA expression level is depicted according to the color scale. Red indicates elevated expression, and green indicates reduced expression. The numbers 2, 0, and − 2 indicate fold changes along the corresponding spectrum. High-BAT group (B1–4). Paired Low-BAT group (A1–4).

**Figure 5:**
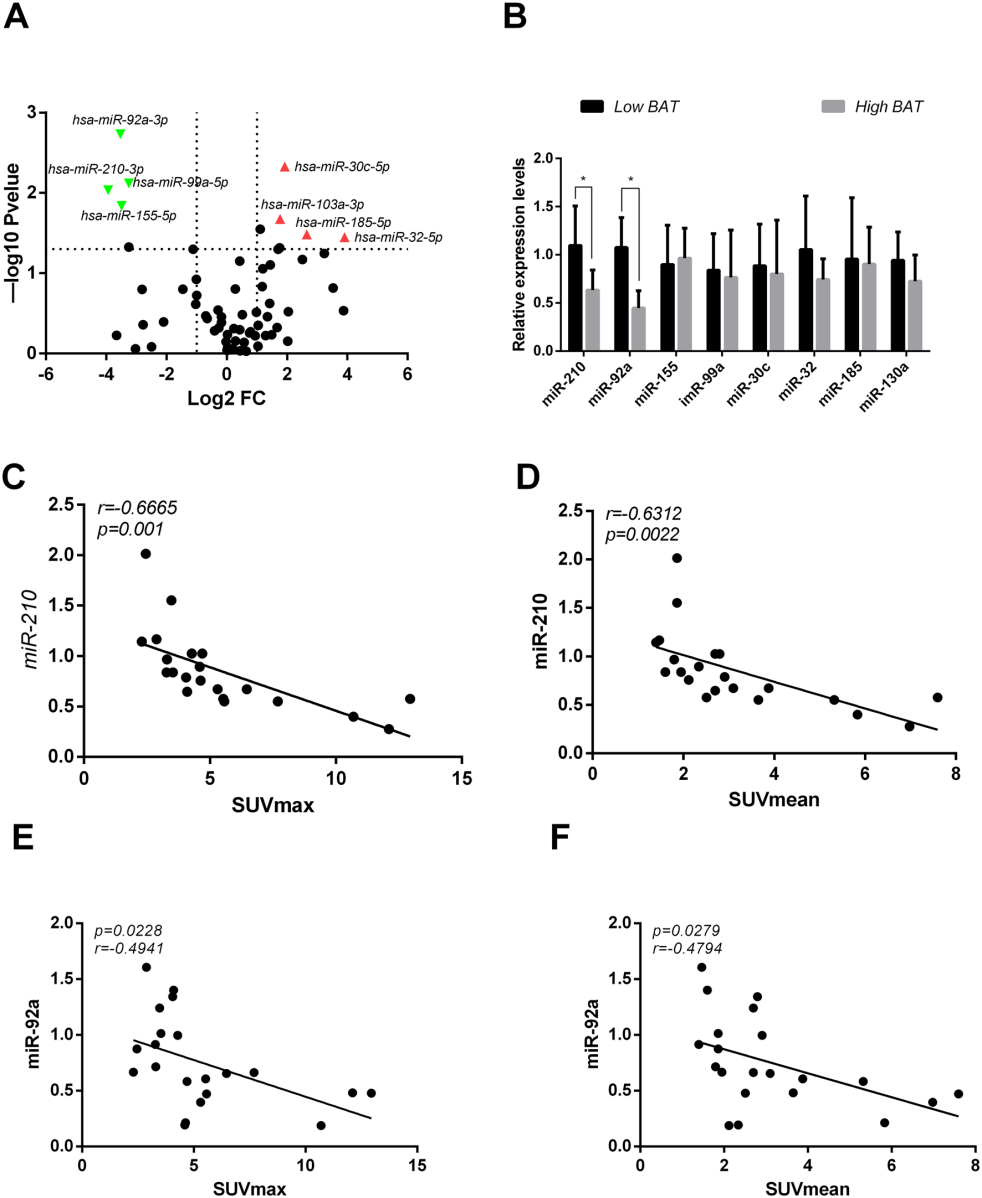
(**A**) Mice serum exosomal miRNA expression profiles in the 2,300 m and 4,300 m groups are shown in the volcano plots as the log transformed fold up- and downregulation values; exosomal miRNAs for which − 1 < log2 (fold change) > 1 and − log10 (p value) > 1.303 were considered significantly differentially expressed. (**B**) Human serum exosomal miRNAs were detected in the high-BAT and low-BAT groups, and the relative quantification was obtained by the ∆∆CT method with normalization to U6 (n = 21 per group, *p ≤ 0.05 indicates statistical significance). The low-BAT group was the control group. (**C**–**F**) The relationship between the relative expression level of miR-210 or miR-92a in human serum exosomes (∆∆CT method vs. U6) and ^18^F-FDG PET/CT brown adipose SUVmax/mean values (Pearson correlation; n = 21; p ≤ 0.05 indicates statistical significance).

Then, to confirm the differential expression of serum exosomal miRNAs between the low-BAT and high-BAT groups, we conducted human MiFinder PCR array testing on eight volunteers. The most abundantly expressed and verified 84 miRNAs in miRbase were probed on the array as candidates, and the expression profiles are shown in the heatmap and the volcano plot (Figs. 4 and 5A). The top significantly differentially expressed miRNAs included miR-92a, miR-30c, miR-99a, miR-210, miR-155, miR-103a, miR-185 and miR-32. Next, we identified various miRNAs from the above results in serum exosomes from all volunteers. Only miR-210 and miR-92a were significantly altered between the high-BAT and low-BAT groups. (Fig. 5B). In addition, a correlation study was performed between the BAT activity indicated by the SUVmax values from PET/CT scanning and the expression levels of the two most frequently identified exosomal miRNA molecules, revealing strong negative associations between them (Fig. 5C–F).

### WAT-derived miR-210 and miR-92a synergistically inhibited adipose browning via the FGFR-1 signaling pathway

**Figure 6:**
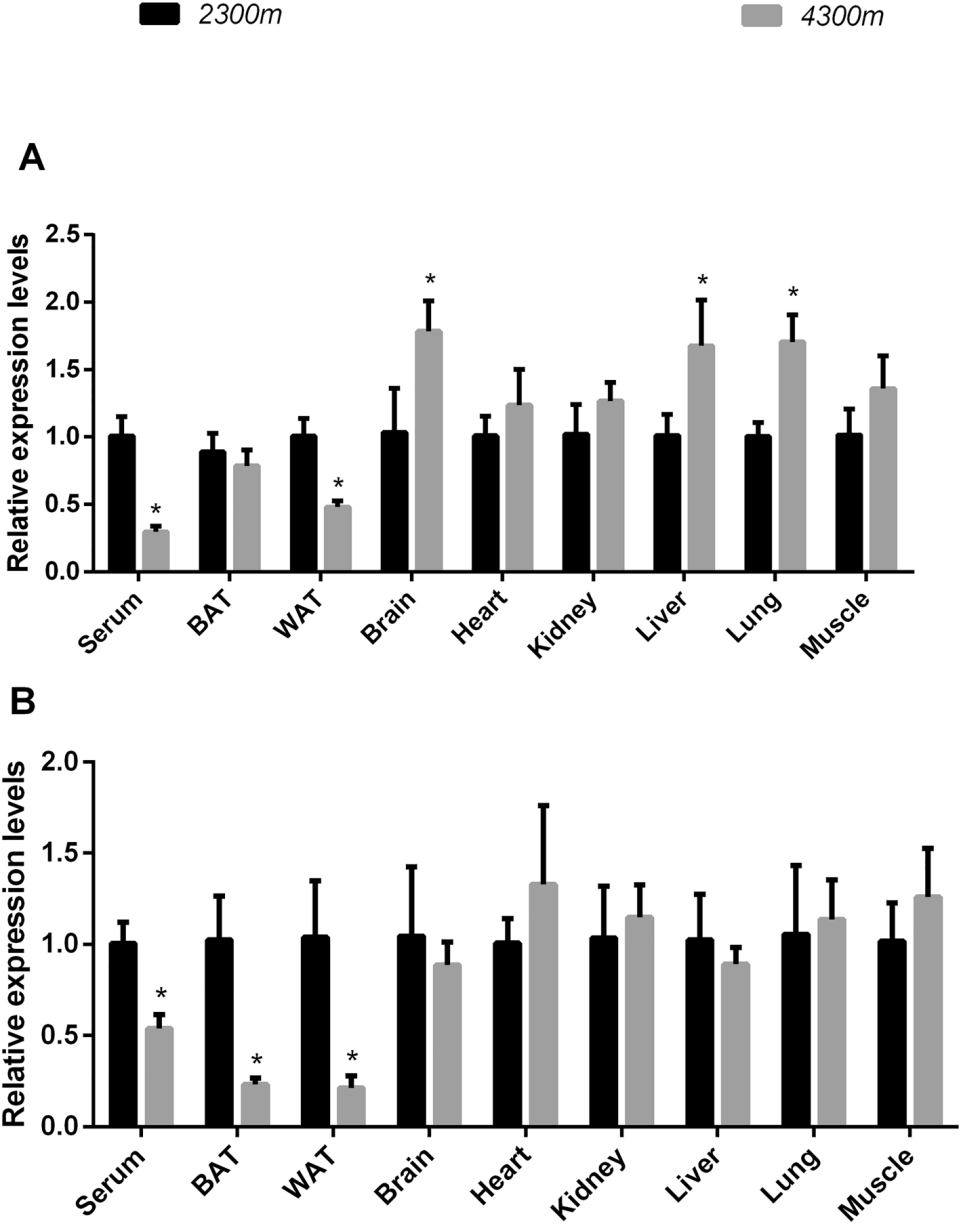
The relative expression levels of miR-210/92a in different tissues of 2,300 m- and 4,300 m-raised mice. (**A**) The relative expression levels of miR-210. (**B**) The relative expression levels of miR-92a. The relative quantification was obtained by the ∆∆CT method with normalization to U6. n = 6 per group; *p < 0.05 indicates statistical significance. The 2,300 m group was used as the control.

Next, the effects of high-altitude-derived exosomal miRNA on BAT activity were analyzed. Given the similar features of BAT activity in humans and rodents in previous studies and since human tissues were unavailable, we utilized mice as an alternative. We selected miR-210 and 92a and validated their expression levels in exosomes from various tissues of mice raised at altitudes of 4,300 m and 2,300 m for 8 weeks. Interestingly, in the 4,300 m group compared with 2,300 m group, the levels of exosomal miR-210 from serum and WAT were decreased, but those from brain, liver, and lung tissue were significantly increased. The levels of the other tissues did not differ between these groups. In addition, exosomal miR-92a expression was downregulated in serum, WAT and BAT but was unchanged in other tissues (Fig. 6).

**Figure 7:**
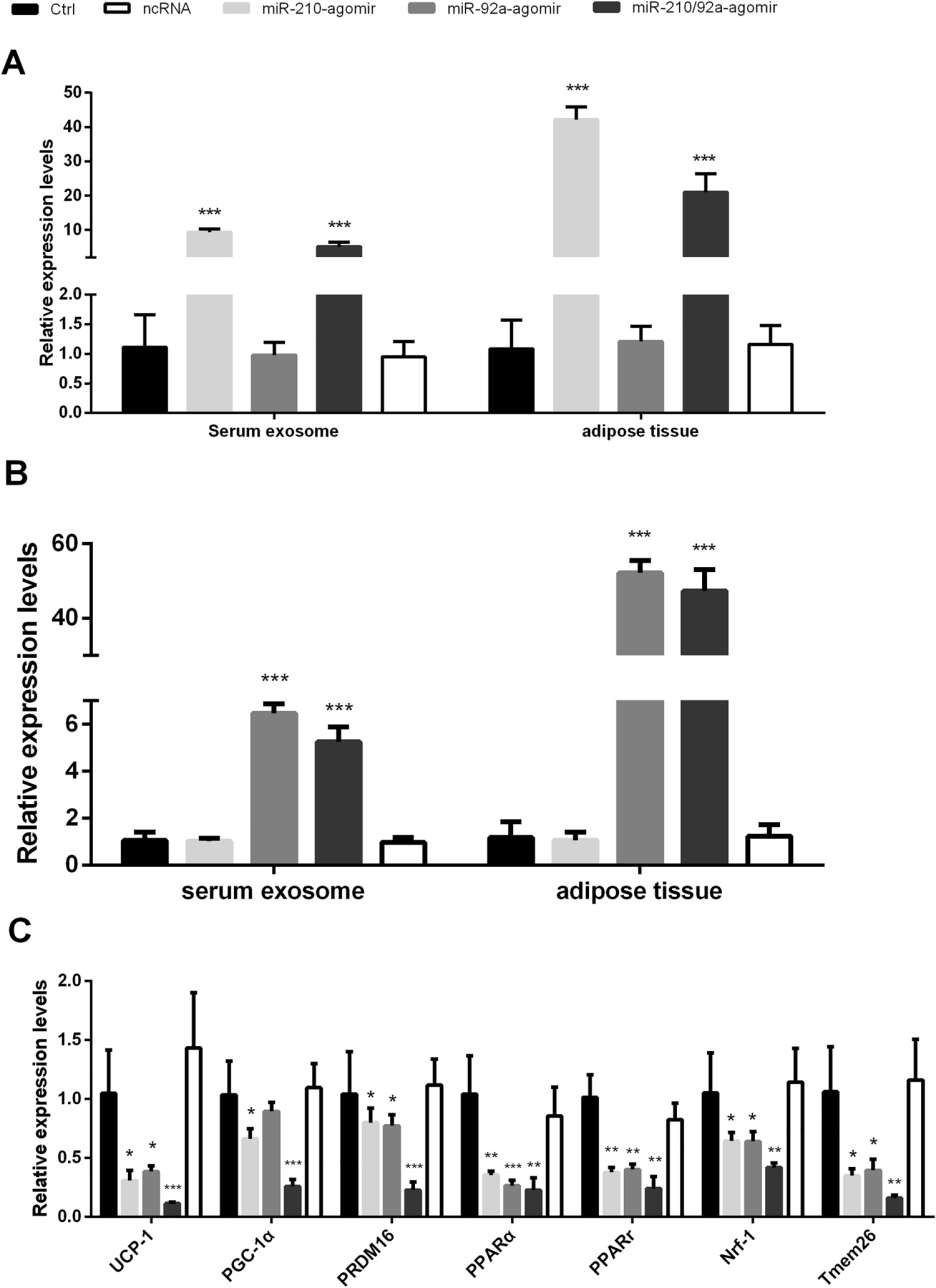
(**A**) The relative expression levels of miR-210 in serum and visceral WAT from the agomir/ncRNA/Ctrl groups of 4,300 m-raised mice. (**B**) The relative expression levels of miR-92a in serum and visceral WAT from the agomir/ncRNA/Ctrl groups of 4,300 m-raised mice. The results were obtained by qPCR analysis. The relative quantification was obtained by the ∆∆CT method with normalization to U6. n = 6 per group. (**C**) Brown adipose-selective genes’ relative expression levels in BAT from the agomir/ncRNA/Ctrl groups of 4,300 m-raised mice. The relative quantification was obtained by the ∆∆CT method compared with 18S rRNA. n = 6 per group (*p ≤ 0.05 indicates statistical significance. **p ≤ 0.01, ***p ≤ 0.001, Student’s *t* test. The Ctrl-(4,300 m) group was used as the control.

**Figure 8:**
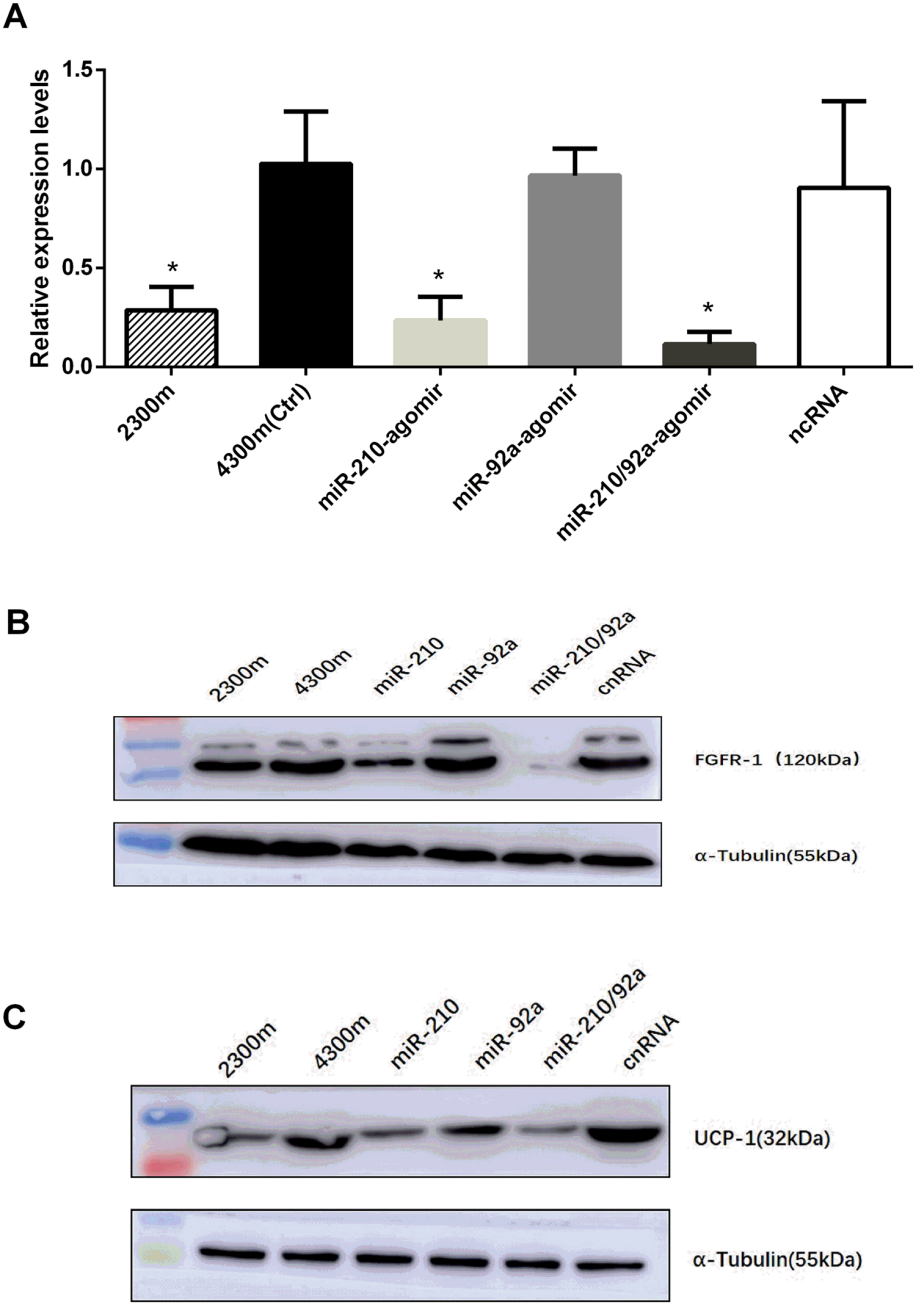
(**A**) The relative expression levels of *Fgfr-1* in BAT from the agomir/ncRNA/Ctrl groups of 4,300 m- and 2,300 m-raised mice. The results were obtained by qPCR analysis. The relative quantification was obtained by the ∆∆CT method with normalization to 18S rRNA. n = 18; *p ≤ 0.05, Student’s *t* test. The Ctrl-(4,300 m) group was used as the control. (**B**,**C**) The protein expression levels of FGFR-1/UCP-1 in BAT from the agomir/ncRNA/Ctrl groups of 4,300 m- and 2300 m-raised mice.

To determine the role of miR-210/92a in vivo under high-altitude hypoxia exposure, we used miRNA-210 and -92a agomirs or a mixture of the two to rescue the expression of miR-210 and -92a in WAT of the 4,300 m group via inguinal subcutaneous fat pad injection. We found that the expression of miR-210 and miR-92a in WAT and serum exosomes was efficiently improved by agomir injection. Compared with the 4,300 m- (Ctrl) and negative control (NC) groups, both the miRNA-210 and -92a agomir injection groups exhibited decreased expression of browning-selective genes. Interestingly, the combing group of both miR-210 and miR-92a-agomirs mixture markedly decreased, which have confirmed that miR210/92a cluster have more efficiently suppressed BAT selective gene expression and protein UCP-1 (Figs. 7, 8C).

Based on previous reports^21–23^ together with online programs TargetScanMouse 6.2 (https://www.targetscan.org/) and miRDB (https://www.mirdb.org/), we have identified* Fgfr-1*, a critical determinant protein of adipose browning factors, as a main predicted target of miR-210, even not established in miR-92a. To validate the* Fgfr-1* expression altered after high-altitude exposure, we analyzed the expression levels of *Fgfr-1* in BAT from 2,300 m- and 4,300 m-raised mice and the expression levels both in mRNA and protein of *Fgfr-1* was significantly higher in 4300 m mice than 2300 m. After injected miR-210-agomir for 4300 m mice, the *Fgfr-1*expression was decreased compared with that in the Ctrl (4,300 m) and NC groups. However, we have not found that any changes in the miR-92a-agomir group. But the expression was the most significantly reduced in miR-210/92a-agomir mixture group, which have compared with injection either miR-210- or miR-92a-agomir alone. (Fig. 8).

### HIF-1α diminished with exposure periods in high-altitude hypoxia

**Figure 9:**
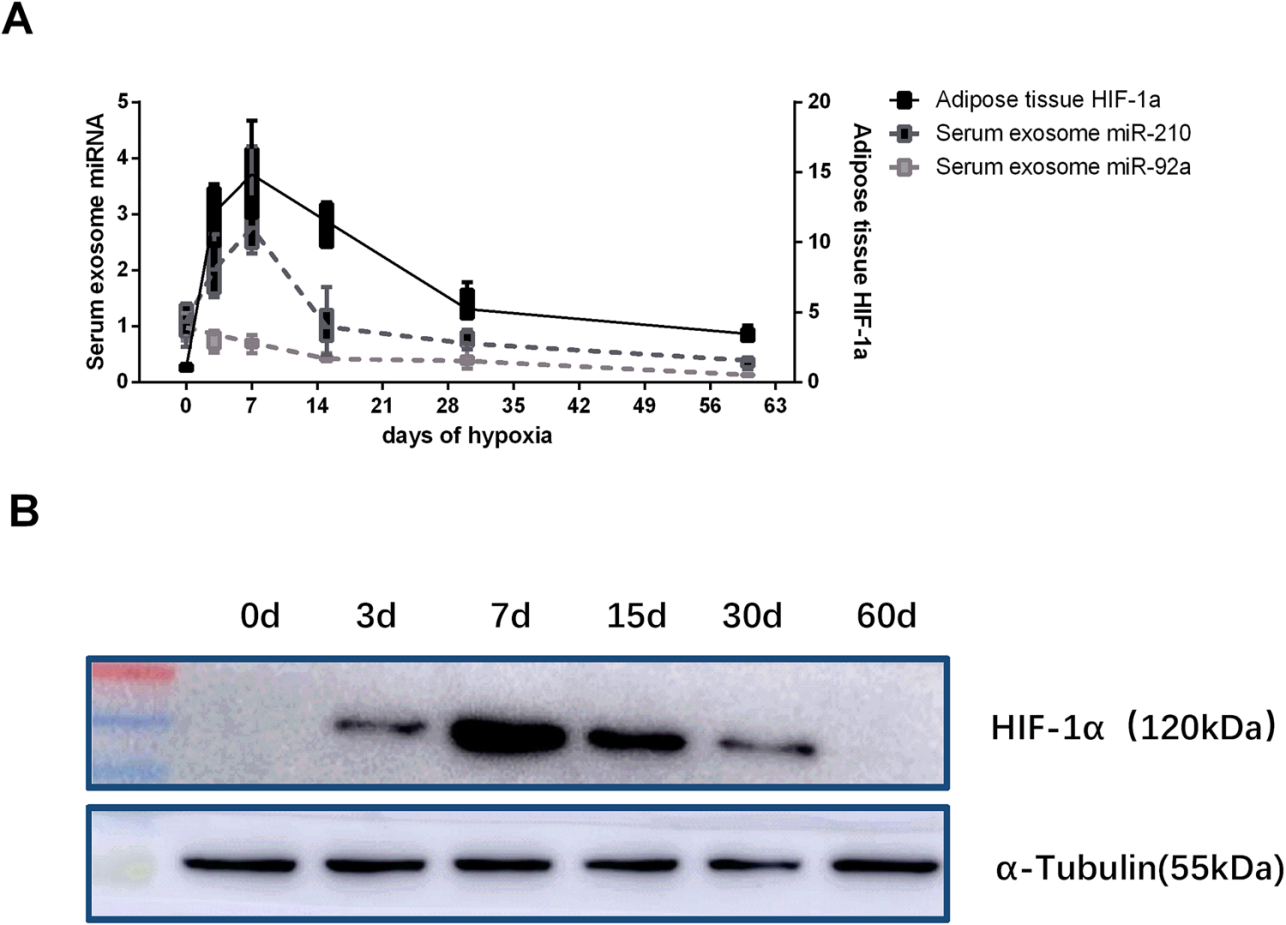
(**A**) Dynamic variation in the relative expression level of *Hif-1α* in visceral WAT and the serum exosomal miR-210/92a with the duration of hypoxia. The left Y axis indicates the relative expression of miR-210/92a, and the right Y axis indicates the relative expression of Hif-1α. (**B**) The dynamic variation in the protein expression levels of HIF-1α as determined by Western blot analysis.

Previous studies indicated that miR-210 is one of the hypoxia-regulated miRNA family members, and have been identified that the augmented parallel expression trends of HIF-1α (hypoxia-inducible factor 1α) with miR-210 under hypoxia^24^. To validate the long-term hypoxia whether have influenced on miR-210 and HIF-1a, we have detected the expression of serum exosomal miR-210 and HIF-1α in WAT at different time points of hypoxic exposure. We found that the expression levels decreased after transit up-forward trends of exosomal miR-210 and WAT HIF-1α in 4,300 m group mice with the time-dependently. However, the expression levels of exosomal miR-92a decreasing after exposure periods. (Fig. 9).

## Discussion

High-altitude hypoxia reduces body fat accumulation and body weight in both humans and rodents. Several mechanisms, including increased sympathetic nerve tone, appetite loss, modulations in genes encoding enzymes involved in glucose metabolism, and suppression of mitochondrial activity, have been proposed to explain the means by which hypoxia-induced metabolic reprogramming can lead to loss of body mass^25^. Additionally, our previous study reported that exposure to hypoxia combined with cold facilitated phenotypic changes in WAT that enhanced browning^15,16^. However, there is no clear evidence of whether hypoxia, as in the case of high-altitude exposure, can affect BAT activity. This study makes clear associations between hypobaric hypoxia and BAT, indicating that the protective effects of hypoxia might be caused by increased BAT activity. Therefore, this study provides novel ideas to the field.

Given that cold exposure can activate BAT and result in the uptake of a large amount of glucose, we recruited volunteers residing at different altitudes and conducted PET/CT scanning according to SUV data. We found that high-altitude exposure-induced BAT activity was increased significantly in subjects living above an altitude of 3,000 m. Therefore, we hypothesized that high-altitude exposure might be involved in the activation of BAT. To assess whether high-altitude hypoxia could potentially impact adipose tissue, we raised mice at altitudes of 2,300 m and 4,300 m. As expected, the mice raised at 4,300 m exhibited signs of elevated BAT activation, such as elevated UCP-1 expression in both WAT and BAT, upregulation of BAT-selective genes in WAT, and enhanced mitochondrial uncoupling. Collectively, the above results supported our hypothesis. BAT dissipates energy, maintains thermoneutrality, and promotes insulin sensitivity; moreover, cold exposure activates BAT in adults (as measured by glucose uptake)^26^ and reduces the risk of obesity and diabetes. Therefore, BAT might contribute to the promising effects of high altitude.

The present study demonstrated changes in BAT levels in humans and mice caused by exosomal miRNA expression levels. In addition, the expression levels of WAT-derived exosomal miRNAs were the same to serum, after injected with miRNAs-angomirs. Interestingly, Ying et al. reported that adipose tissues can release an abundance of exosomes that crosstalk with macrophages in both the remote liver and neighboring adipose tissue to affect insulin sensitivity, thus regulating metabolic disease^27^. Moreover, WAT-derived exosomes have already been demonstrated to be powerful cell–cell communication vehicles via their cargo of exosomal miRNAs, which interfere with global metabolism^28^. In addition, WAT plays an important role in mediating miRNA expression levels by manipulating Dicer activity, and many exosomal miRNAs have been found to be differentially expressed in lean and obese individuals, and these miRNAs have been demonstrated to originate from WAT^29^. A few previous studies indicated miRNAs can be packaged into and transported via exosomes, have profound effects on BAT activity or as biomarkers, Such as miR-103, miR-146b, miR-155 and miR-99a^30^. This finding is consistent with our results showing similar expression in exosomes, but these results were not seen in the mouse models in this study, possibly because they are not hypoxia-sensitive or are not affected by hypobaric hypoxia. However, in contrast, our study showed elevated BAT activity accompanied by significantly decreased expression levels of miR-210 and miR-92a.

miR-210 is a specific target of *Hif-1α*^31^ and exosomal miR-210 from hypoxic cells can be transferred to various types of recipient cells and involved in various processes, including the cell cycle, mitochondrial oxidative metabolism, angiogenesis, the DNA damage response, and cell survival^32^. However, few studies have shown the expression and function of *Hif-α* in tissues under long-term exposure to environmental hypoxia. In this study, we found that miR-210 levels were significantly decreased in the high-BAT group compared with the low-BAT group. Similar results were also observed in mice exposed to chronic hypoxia, in which the activity of mitochondria in BAT indicated that oxygen consumption increased in complex I activity. In addition, the expression levels of *Hif-1α* in WAT has suppressed significantly after 8 weeks exposure to hypobaric hypoxia. Furthermore, miR-210 had significantly lower in the serum- and WAT-derived exosomes of mice after long-term hypoxia exposure. which can be attributed to the unique hypoxia-sensing and HIF signaling pathways and exosome secretion mechanisms in adipose tissues. Our study also showed that *Fgfr-1* in BAT has increased significantly in 4,300 m mice, but after manipulatively increased the miR-210 by locally injected agomir, *Fgfr-1* have noticeably suppressed. Due to effective stimulator for improving BAT activity, FGF-21 is a critical protein to influence on metabolic homeostasis^33^. FGFR-1, the most affinity receptor with FGF-21, is a family member of the tyrosine kinase receptors FGFR isoforms, normally expressed on WAT, BAT and hypothalamus^34^. Based on the previous reports^35^, FGF-21/FGFR-1 signals induced BAT activity majorly caused by regulations of PGC-1a and UCP-1 pathway. Moreover, FGFR-1, a major target downstream, can be negatively modulated by miR-210^23^. FGFR-1 played important role in hypoxia microenvironment, with involved in development, proliferation, and angiogenesis in adipocytes^36^. Consistently with the current study, BAT activity was enhanced by specifically promoted FGFR-1 on mice adipose tissues^36^, but *Fgfr-1* KO mice shown the opposite effects^37^. Totally, this study indicated that high-altitude hypoxia activated BAT through attenuated WAT secreted exosomal miR-210, which enhanced the FGFR-1 expression in BAT.

Interestingly, we found that the expression level of FGFR-1 in BAT was not changed, but UCP-1 expression was significantly decreased, in the 4,300 m raised mice with miR-92a-agomir-injected groups. Furthermore, the expression level of FGFR-1 was decreased more obviously in the miR-210/92a agomir-injected groups compared with the group injected with the miR-210 agomir alone. It indicated that miR-92a increased the inhibitory effect of miR-210 on FGFR-1, miR-210/92a cluster synergistically increased the inhibition of BAT activity. Furthermore, miR-92a did not directly decrease BAT activity through the FGFR-1 pathway and that other mechanisms may be involved.

Notably, this study showed that the expression level of serum exosomal miR-92a increased in subjects with high BAT and in mice raised under hypoxic conditions. Similarly, miR-92a is an adipose-derived miRNA and exhibited low expression levels in serum exosome and WAT from high-altitude-raised mice. The significant biomarker for BAT was miR-92, according to the evidence from Chen Y^38^ together with the angiogenesis-modulating effects of miR-92a in adipose tissues. Decreased miR-92a expression might promote angiogenesis in BAT after hypoxia, resulting in enhanced blood supply to local adipose tissues followed by an increase in browning activity. Furthermore, the similar responses of miR-92a and miR-210 after exposure to hypoxia might indicate the involvement of miR-92a in both hypoxic and metabolic signaling.

Our study has some limitations. First, we conduct PET/CT scans only in high-altitude populations. We utilized all subjects to study exposure to hypoxia at different altitudes without the sea level control group. This methodology could have influenced the BAT results. Furthermore, no data are available for sea level human populations or mice raised at sea level. Second, the small sample size for the collection of PET/CT data was due to insufficient funding along with expensive testing fees and radiation exposure risks of PET/CT to healthy people in the clinic. Finally, the specific genetic manipulations of miRNAs have not been validated at the cellular level. Such validation needs to be conducted in future studies.

## Methods

### Study approval

The human study was approved by the Ethics Committees of Qinghai University (No. QHUM-20171101HM) and Qinghai Provincial People’s Hospital (QHPH-20171122). All subjects provided written informed consent prior to any study procedure. All procedures were conducted in accordance with the principles of the Declaration of Helsinki.

### Statistics

General parameters for subject characteristics are presented as the mean ± s.d. Statistical differences were assessed by applying Student’s *t* test (unpaired, two-tailed). Pearson correlation was used in correlation analysis. GraphPad Prism 6 or Excel software (Microsoft Office) was applied to calculate p values. The level of statistical significance was set at p ≤ 0.05.

### PET/CT scanning and baseline measurements

We recruited 21 individuals of Han Chinese descent living at different altitudes on the Qinghai-Tibet Plateau. The details are shown in Supplement Table 1. From each volunteer, after 8 h of fasting, 15 ml of venous whole blood was collected for the measurement of serum levels of triglycerides (TG), cholesterol (CHOL), high-density lipoprotein cholesterol (HDL-C), low-density lipoprotein cholesterol (LDL-C), and so on. Serum samples were isolated within 1 h, and exosomes were then purified from each serum sample. The general features of exosomes isolated from serum in the current study were confirmed by FACS analysis of surface CD63 and TEM, as reported previously. Prior to PET/CT scanning, the height and weight, along with the waist and hip circumference, were measured. After 8 h of fasting, ^18^F-FDG was injected intravenously at a dose of 0.1 mCi per kilogram of body weight. A full-body PET/CT scan was performed after a rest period at 4 °C for 60 min. BAT activity was measured from static PET scans as the mean and maximal ^18^F-FDG uptake in BAT. The investigated regions were manually outlined using a threshold of 1.5 SUV and Hounsfield units between − 10 and − 180 to define BAT. The metabolic and PET characteristics of the recruited human subjects are shown in Table 1.

### Animal models

Six-week-old male C57BL/6J mice were housed in a temperature-controlled environment on a 12:12 h light: dark cycle at altitudes of 2,300 m and 4,300 m, separately. The temperature and humidity were controlled to remain within 22–25 °C and 50–60%, respectively. The water and food supply were unrestricted. To determine the influence in serum exosomal miRNA and adipose tissue HIF-1α expression that may occur at long-term hypoxia exposure, mice were euthanized at six different stages (hypoxia exposure day 0, 3, 7, 15, 30 and 60). And then the serum and tissues were taken and frozen in liquid nitrogen within 30 s for the next study. For in vivo experiments, we delivered miR-210 or miR-92a agomirs (20 nmol/mice) or scrambled negative control sequences (GenePharma, Shanghai, China) to the 4,300 m-raised mice though inguinal subcutaneous fat pad injection. The injections were performed four times at three-day intervals. After the final injection, the mice were housed under the same conditions described above. Four weeks after the final injection, serum and tissues were collected for the next experiment. All experiments were conducted in strict accordance with the National Institutes of Health Guide for the Use of Laboratory Animals. All efforts were made to minimize suffering. The general parameters of mice data are shown in Table 2.

**Table 2:** The general parameters of mice data.

	Total (n = 40)	2,300 m (n = 20)	4,300 m (n = 20)	p
Weight (g)	25.12 ± 1.65	26.03 ± 1.5	24.22 ± 1.3	0.000*
RBC (10^12^/l)	8.91 ± 0.837	8.78 ± 0.87	9.08 ± 0.79	0.355
HGB (g/l)	140 ± 13.6	133 ± 10.7	150 ± 10.5	0.000*
HCT%	43.2 ± 4.19	41.3 ± 3.76	45.6 ± 3.5	0.000*
WBC (10^9^/l)	3.68 ± 2.14	3.04 ± 1.61	4.47 ± 2.31	0.086

### Exosome isolation, fixation, and identification

Venous blood was collected from humans and mice, and serum was separated for the next experiment. Fresh tissue materials (100 mg), such as adipose, liver, heart, and brain tissue, were split into small pieces and soaked in 1 ml of PBS. Samples were incubated for 4 h at room temperature for complete exosome release, and the PBS was then collected for the next experiment^27^.

A total of 250 µl of murine or human serum and 1 ml of exosome-rich PBS were isolated using ExoQuick exosome precipitation solution (#: EXOQ5A-1, SBI) following the manufacturer’s protocol. For exosome loading, exosome preparations were isolated and diluted with PBS to a final volume of 100 µl and prepared for electron microscopy and other experiments. The exosomes were resuspended in 50–100 μl of 2% PFA, and 5 μl of the exosomal suspension was added to a Formvar/carbon-loaded copper mesh and washed with PBS. In all steps, the Formvar membrane was kept moist, and the other side was kept dry. The copper mesh was placed on 50 μl of 1% glutaraldehyde droplets for 5 min and washed 8 times in 100 μl of ultrapure water for 5 min. The copper mesh carrying the exosomes was placed on 50 μl of uranyl oxalate droplets (pH 7) for 5 min and then on 50 μl of methylcellulose droplets for 10 min. The excess liquid on the filter paper was aspirated, and the mesh was dried in air for 5–10 min. All of the above operations were carried out on ice. Electron micrographs were acquired at 80 kV. To determine the exosomal particle sizes, the concentration was assessed using NTA. An NTA device (ZetaView^®^, Particle Metrix, Germany) was used to measure the particle size, concentration, and zeta potential of exosomes by analyzing the particle traces. To evaluate exosome marker proteins, exosomes were lysed with RIPA buffer, and total protein was separated by SDS-PAGE using 12% polyacrylamide gels and transferred to a PVDF membrane, which was blocked with 5% BSA for 1 h and incubated with primary antibodies at 4 °C overnight. Antibody binding was detected with a chemiluminescence reagent (Thermo Fisher Scientific). Antibodies included rabbit anti-CD9 (#: ab 92726, Abcam), diluted 1:2000; rabbit anti-CD63 (#: ab 217345, Abcam), diluted 1:1,000; and goat anti-rabbit IgG (#: SA00001-2, Proteintech), diluted 1:1,000.

### mRNA/miRNA PCR and MiFinder PCR array

mRNA was isolated from mouse tissues using an RNAsimple Total RNA Isolation Kit (#: DP419, TIANGEN China Beijing). Reverse transcription and fluorescence quantitative PCR of tissue mRNA were performed with FastKing gDNA Dispelling RT SuperMix (#: KR118, TIANGEN China Beijing) and a SuperReal PreMix Kit (SYBR Green) (#: FP215, TIANGEN China Beijing).

For isolation of serum or tissue exosomal miRNA, 400–600 µl of exosome extract was obtained from mice or humans, and miRNAs were isolated using a SeraMir Exosome RNA Purification Kit (#: RA806A-1, SBI) following the manufacturer’s protocol. Reverse transcription and fluorescence quantitative PCR of all miRNA samples was performed with a miRcute Enhanced miRNA cDNA First Chain Synthesis Kit (#: KR211, TIANGEN China Beijing) and a miRcute Enhanced miRNA Fluorescence Quantification Kit (SYBR Green) with reverse universal primers (#: FP411, TIANGEN China Beijing). A miScript™ miRNA PCR Array Human Hypoxia Signaling (#: MIHS-121Z QIAGEN Germany) was analyzed according to the manufacturer’s protocol. The primer sequences are provided in Table 3.

**Table 3:** Primer sequences for genes.

mRNAs/miRNAs	5′ → 3′
18S	
Forward	TTGACGGAAGGGCACCACCAG
Reverse	GCACCACCACCCACGGAATCG
Ucp-1	
Forward	CAGAGGTCGTGAAGGTCAGAATGC
Reverse	GGACATCGCACAGCTTGGTACG
Ppargc1α	
Forward	CCGAAGACACTACAGGTTCCATAG
Reverse	GGGAGGGAGAGAGGAGAGAGG
Prdm16	
Forward	CAACAAAGAGAAGCCGTTCAAG
Reverse	TTTCGGATCTCGGAGAAGTAAG
Pparα	
Forward	GAGCTGCAAGATTCAGAAGAAG
Reverse	GAATCTTTCAGGTCGTGTTCAC
Pparγ	
Forward	TGTGGACCTCTCCGTGATGG
Reverse	GGTTCTACTTTGATCGCACTTTGG
Nrf1	
Forward	CCACGTTGGATGAGTACACG
Reverse	GCACCACATTCTCCAAAGGT
Tfam	
Forward	TCAGGAGCAGCAGGCACTACA
Reverse	CTGAGCTCCGAGTCCTTGAACAC
Tmem26	
Forward	GTGGTGTGGACGTGGAGTATGC
Reverse	GTAGATCATCAGGACGAGGCGC
Hif-1α	
Forward	CCACCACAACTGCCACCACTG
Reverse	TGCCACTGTATGCTGATGCCTTAG
Fgfr-1	
Forward	TTGCCAGACCAAGAAGAAGCCATG
Reverse	GTCCTTGTCACCACTGCGTTCTC
has-/mmu-miR-210-3p	
Forward	CUGUGCGUGUGACAGCGGCUGA
has-/mmu-miR-92a-3p	
Forward	UAUUGCACUUGUCCCGGCCUG
hsa-miR-155-5p	
Forward	UUAAUGCUAAUUGUGAUAGGGGU
hsa-miR-99a-5p	
Forward	AACCCGUAGAUCCGAUCUUGUG
has-miR-30c-5p	
Forward	UGUAAACAUCCUACACUCUCAGC
hsa-miR-32-3p	
Forward	CAAUUUAGUGUGUGUGAUAUUU
hsa-miR-185-5p	
Forward	UGGAGAGAAAGGCAGUUCCUGA
hsa-miR-130a-3p	
Forward	CAGUGCAAUGUUAAAAGGGCAU

### Western blot analysis and histomorphology

The method used for Western blot analysis of tissue proteins was the same as that used for exosome markers. Antibodies included rabbit anti-UCP-1 (#: ab10983, Abcam), diluted 1:5,000; rabbit anti-FGFR-1 (#: 9740, CST), rabbit anti-HIF-1α (#: 36169, CST), α-Tubulin Rabbit Polyclonal Antibody (#: AF0001, Beyotime), diluted 1:1,000; and goat anti-rabbit IgG (#: SA00001-2, Proteintech),diluted 1:1,000. Immunohistochemical staining was performed according to standard methods. The antibody used was rabbit anti-UCP-1 (#: ab10983, Abcam), diluted 1:500.

### Mitochondrial respiration assay

BAT mitochondrial respiration rates were determined by measuring the OCR with a high-resolution mitochondrial respirometer (Oxygraph-2k). In brief, 5 mg of pure adipose tissue was isolated in 500 μl of precooled MiR05 respirator (110 mM sucrose, 60 mM K-lactobionate, 0.5 mM EGTA, 3 mM MgCl_2_, 20 mM taurine, 10 mM KH_2_PO_4_, 20 mM HEPES (pH 7.1) and 0.1% BSA at 30 °C, homogenized, and added to the chamber of the high-resolution mitochondrial respirometer. The temperature in the chamber was maintained at 37 °C. Pyruvate (5 mM), malic acid (5 mM), and sodium glutamate (10 mM) were used to measure the respiration rate according to substrate-uncoupler-inhibitor-titration (SUIT) protocols. After baseline stabilization, the state 4 respiration rate (CI-LEAK) of respiratory chain complex I was obtained. ADP (10 mM), cytochrome C (10 μM), succinic acid (10 mM), FCCP (0.25–1.5 μM), and rotenone (0.5 µM) were sequentially added. The mitochondrial respiratory chain complex I state 3 respiration rate (CI-OXPHOS), respiratory chain complexes I + II state 3 respiration rate (CI + II-OXPHOS), and electron transport capacity (CI + II-ETS), as well as the electron transport capacity (CII-ETS) and uncoupling ability of respiratory chain complex II were measured under the condition of intact mitochondrial inner membranes.

## Supplementary information

Supplementary Information.
