# Supplementary material for: Adipose-derived exosomal miR-210/92a cluster inhibits adipose browning via the FGFR-1 signaling pathway in high-altitude hypoxia
Source: Sci Rep. 2020 Sep 1;10:14390. doi: 10.1038/s41598-020-71345-8 (PMC7463015; doi:10.1038/s41598-020-71345-8)

Supplemental Information

Adipose-derived exosomal miR-210/92a cluster inhibits adipose browning via the FGFR-1 signaling pathway in high-altitude hypoxia

Yifan Zhang [1,2,3,4], Kang Song [1,2,3,4], Gang Qi[4], Ranran Yan[1], Yanqing Yang[4], Yan Li[4], Shunjuan Wang[1,2,3,4], Zhenzhong Bai[1,2,3 *], Ri-li Ge [1,2,3 *]

1.Research Center for High Altitude Medicine, Qinghai University Medical College. Qinghai, Xining, 810001, PR China
2.Key Laboratory of High-altitude medicine (Qinghai University), Ministry of Education. Qinghai, Xining, 810001, PR China
3.Key Laboratory for Application of High-Altitude Medicine in Qinghai Province. Qinghai, Xining, 810001, PR China
4. Qinghai Provincial People's Hospital. Qinghai, Xining, 810007, PR China

ZB and RG *corresponding author
geriligao@hotmail.com,
2003980001@qhu.edu.cn

Author Contributions statement

BZ and GR conceived and designed the experiments, BZ wrote the manuscript, ZY analysed, interpreted results. ZY, SK, and YR performed animal experiments and measurement of parameters. SK performed mitochondrial respiration measurement with O2K. QG, YY, LY, and WS run PET-CT scan. GR contributed to proof-reading and correction of manuscript.

Acknowledgements

This work was supported by the National Natural Science Foundation of China (31571231);
Qinghai International Collaboration Program (2015-HZ-807);
Guiding Program of Qinghai Provincial Health Commission (2019-wjzdx-04).

Additional information

Conflict of Interest

None of the other authors declare a relevant conflict of any financial or non-financial interest.

Supplement table1: General information on all subjects

NO.	Gender	Age（years）	Altitude（m）	City	Length of residence(years)
1	F	38	3696	Zeku	17
2	M	34	4292	Maduo	10
3	M	36	2443	Huzhu	36
4	F	32	2443	Huzhu	32
5	F	36	2260	Xining	36
6	M	32	3730	Maqin	12
7	M	31	4276	Zhiduo	10
8	M	33	2118	Pingan	33
9	M	35	3292	Gangcha	15
10	F	36	3696	Tianjun	36
11	F	37	4276	Zhiduo	15
12	F	37	3696	Tianjun	30
13	F	38	3730	Maqin	15
14	M	35	2260	Xining	35
15	M	39	2260	Xining	28
16	M	34	2118	Minhe	34
17	M	33	4276	Qumalai	10
18	F	33	2260	Xining	33
19	F	36	2260	Xining	20
20	F	31	3696	Banma	13
21	M	32	2260	Xining	20

Supplement figures

Figure S1 （The Original data of Figure. 2 D1）

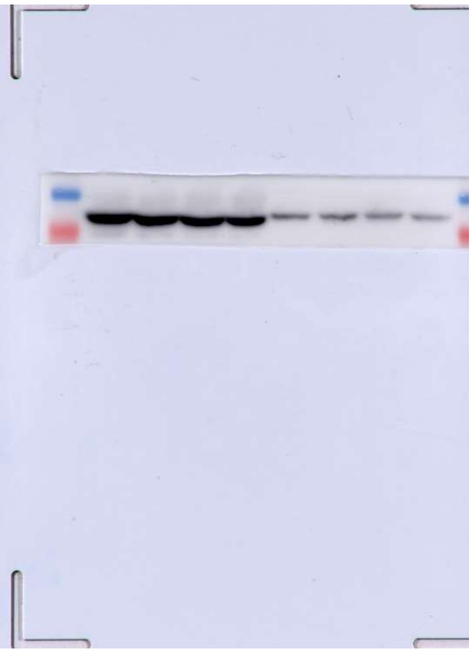

Figure S2 （The Original data of Figure. 2 D2）

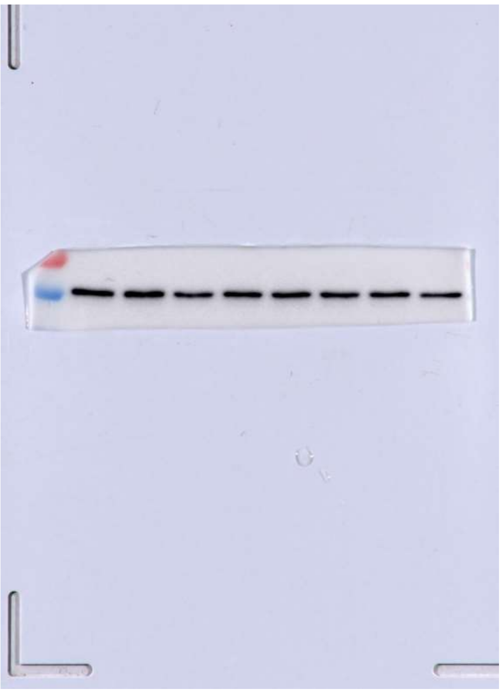

Figure S3 （The Original data of Figure. 3 A1）

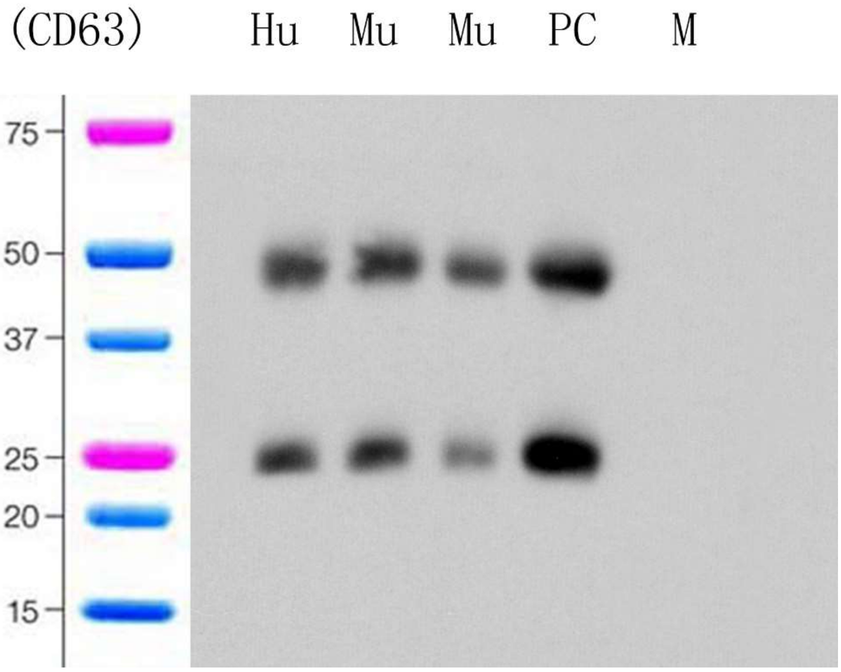

Figure S4 （The Original data of Figure. 3 A2）

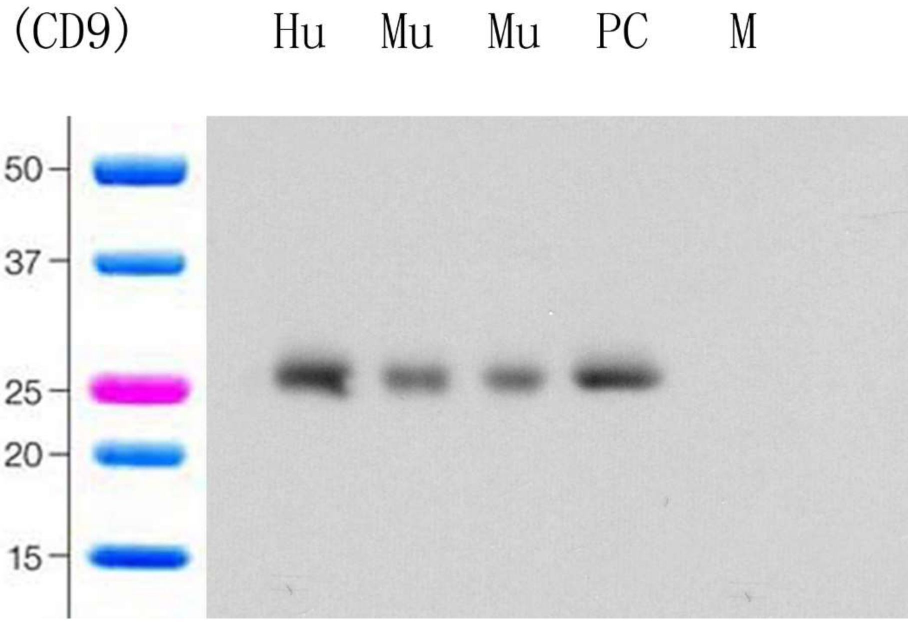

Figure S5 （The Original data of Figure 8 B1）

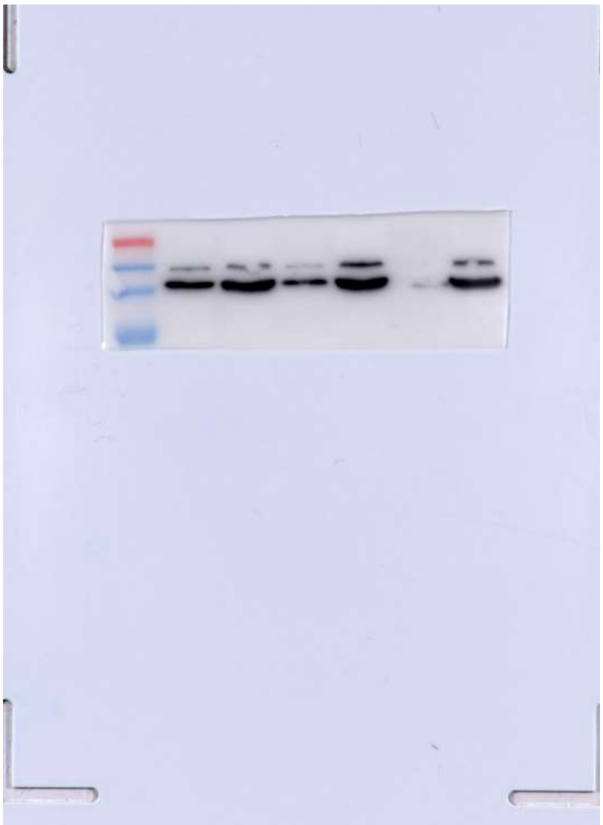

Figure S6 （The Original data of Figure 8 B2）

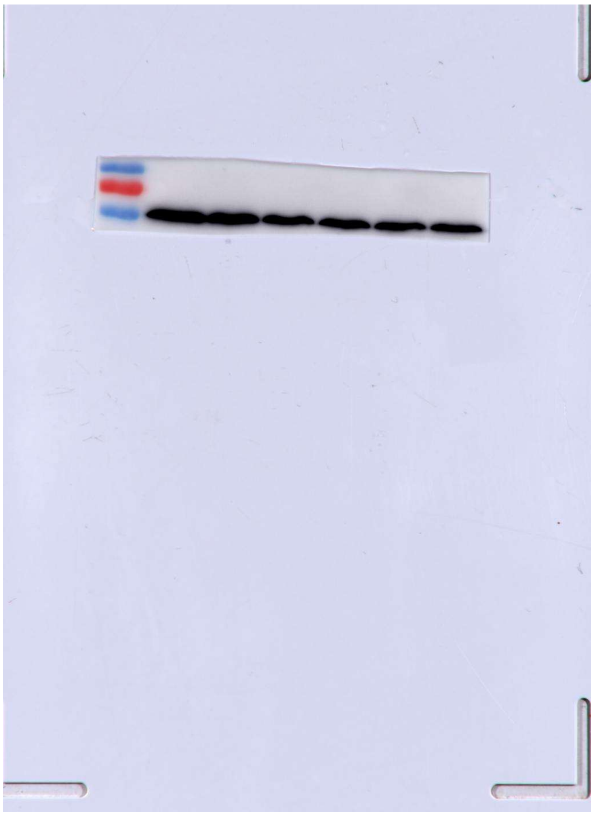

Figure S7 （The Original data of Figure 8 C1）

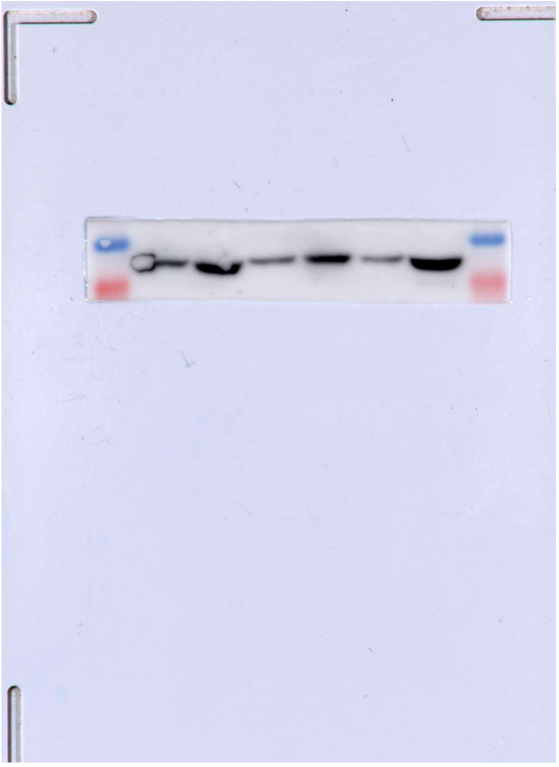

Figure S8 （The Original data of Figure 8 C2）

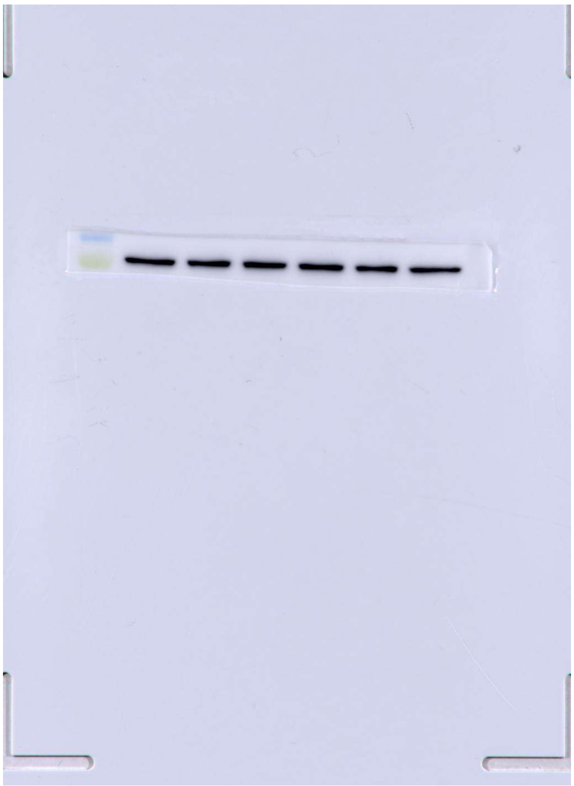

Figure S9 （The Original data of Figure 9 B1）

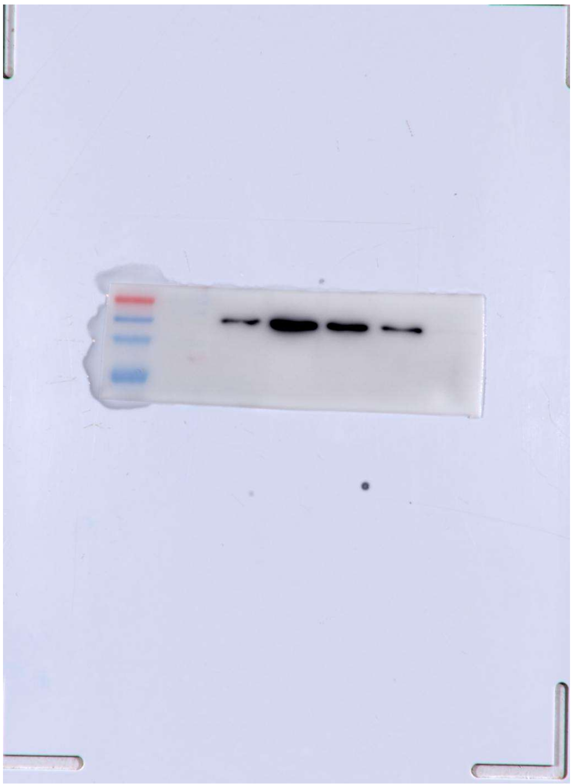

Figure S10 （The Original data of Figure 9 B2）

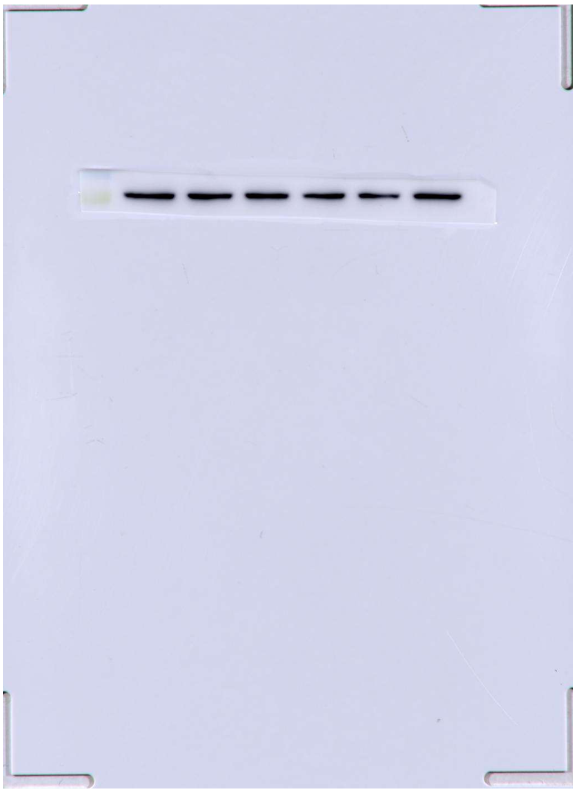

Supplement: Supplementary file 1 — Supplementary Information. [file 41598_2020_71345_MOESM1_ESM.pdf]
